# Tele-pulmonary rehabilitation

**Published:** 2012-06-15

**Authors:** Christine McClusky, Morag Barrow

**Affiliations:** NHS 24, Scottish Centre for Telehealth and Telecare, UK; NHS 24, Scottish Centre for Telehealth and Telecare, UK

**Keywords:** pulmonary rehabilitation, access, effective, innovation

## Abstract

**Background and objectives:**

COPD Guidelines recommend that programmes of pulmonary rehabilitation (PR) are tailored to individuals to improve symptoms, quality of life and self management. Accessing PR is dependent on travelling to classes. Many people, who live in rural areas, are socially isolated, or who live with severe disease that reduces mobility, are unable to travel far. Patients in Scotland were invited to participate in ‘hub and spoke’ PR programmes of physical activity, disease education and support, linking sites using video conferencing technologies. The key objectives for the project were: improved health, well-being and empowerment; increased physical fitness; reduced exacerbations and anxiety; improved quality of life; greater independence; reduced social isolation through peer support; fewer emergency admissions.

**Methodology:**

Building on a feasibility study from NHS Tayside, a steering group, including patient partners and voluntary organisations, was established. Clinical outcomes were measured before and after the programmes. Two measures were used: walking tests (Six minute walking distance or incremental shuttle walking distance) and the Chronic Respiratory Questionnaire (CRQ). The CRQ consists of four sections—Dyspnoea (breathlessness), Fatigue, Emotional Function and Mastery (sense of control over the disease process). A satisfaction questionnaire was drawn up for the project, incorporating a Client Satisfaction Questionnaire, together with some specific questions relating to the video conferencing experience. Digital patient stories were captured (www.patientvoices.org.uk/sth.htm). The innovative use of pc based video conferencing systems for clinical use was explored.

**Results:**

Two hundred and twenty-six patients: 110 conventional classes; 110 telelinked classes; 6 conventional class with tele-education. Mean age 67 years. Mean improvement in walking distance 37%. Mean improvement in Chronic Respiratory Questionnaire domain scores: 2.7 dyspnoea; 2.7 fatigue; 2.4 emotional function; 1.5 mastery. Thirty percent additional capacity at each tele PR class. Cost per patient decreased due to increased throughput.

**Conclusions and future developments:**

The delivery of pulmonary rehabilitation using telelinks is at least as good as a traditional model and is acceptable to patients. More patients able to benefit from PR; services delivered closer to home; contributes to peer support and cost efficient. PC based video conferencing technologies works well and is more flexible and portable. The teams are now working on domiciliary (home based) tele-pulmonary rehabilitation, allowing access to patients who are either socially isolated or who are at a more severe stage of the disease. These patients have previously not had consistent, equitable access to rehabilitation, despite evidence showing that functional benefits can still be achieved. We will be able to report on early findings from this work at the conference.

